# Antioxidant Treatment Reverts Increased Arterial Basal Tone and Oxidative Stress in Nephrectomized (5/6) Hypertensive Rats

**DOI:** 10.1155/2013/863067

**Published:** 2013-03-13

**Authors:** Rodrigo O. Marañón, Claudio Joo Turoni, Maria Sofia Karbiner, Nicolas Salas, Maria Peral de Bruno

**Affiliations:** ^1^Departamento de Fisiología, INSIBIO-Universidad Nacional de Tucumán, Balcarce 32, 4000 Tucumán, Argentina; ^2^Department of Physiology and Biophysics, University of Mississippi Medical Center, 2500 North State Street, Jackson, MS 39216, USA

## Abstract

Nonischemic 5/6 nephrectomized rat (NefR) is a model of chronic kidney disease. However, little is known about vascular dysfunction and its relation with hypertension in NefR. *Aims*. To evaluate possible alterations of endothelial function, NO-bioavailability, and basal tone in aorta from NefR and the role of oxidative stress. Sprague Dawley rats were divided into sham rats (SR), NefR, and NefR treated with tempol (NefR-T). Mean arterial pressure (MAP) and renal function were determined. In isolated aortic rings the following was measured: 1-endothelial function, 2-basal tone, 3-NO levels, 4-membrane potential (MP), and 5-oxidative stress. NefR increased MAP (SR: 119 ± 4 mmHg; *n* = 7; NefR: 169 ± 6; *n* = 8; *P* < 0.001). Tempol did not modify MAP (NefR-T: 168 ± 10; *n* = 6; *P* < 0.001). NefR showed endothelial dysfunction, increased basal tone and decreased NO levels (SR: 32 ± 2 nA; *n* = 7, NefR: 10 ± 2; *n* = 8; *P* < 0.001). In both in vitro and in vivo tempol improves basal tone, NO levels, and MP. Oxidative stress in NefR was reverted in NefR-T. We described, for the first time, that aorta from NefR presented increased basal tone related to endothelial dysfunction and decreased NO-bioavailability. The fact that tempol improves NO-contents and basal tone, without decrease MAP, indicates that oxidative stress could be implicated early and independently to hypertension, in the vascular alterations.

## 1. Introduction

It is known that, in the chronic kidney disease (CKD), the endothelial dysfunction could be cause and/or consequence of the kidney damage. Nonischemic 5/6 nephrectomized rat (NefR) is a model used to evaluate the evolution of renal abnormalities in CKD [1]. A lot of evidence has reported that the decrease of renal mass is a cardiovascular risk factor [2]. Recently the concept that reduced renal mass isassociated with low birth weight as a cardiovascular risk factor has gained importance. These suggestions have been supported by both clinical [3] and experimental evidence [4]. Xie et al. showed that rats with low birth weight impaired renal function and developed hypertension.

In NefR, the impact of the loss of renal mass, its relationship to vascular dysfunction, and the development of hypertension have been little studied. It has been shown, in rats, that the renal mass reduction (2/3) associated with ischemia of contralateral kidney was able to induce hypertension [5]. In this situation, the hypertension has been explained by renin-angiotensin system activation. However, the implicated mechanisms in the development of hypertension in NefR without renal ischemia are unclear. In this model, to the best of our knowledge, arterial basal tone, endothelial function, and nitric oxide (NO), which are known to be involved in the regulation of arterial pressure have not been evaluated in a comprehensive way. In NefR, some authors did not find hypertension [6, 7]; however other authors found elevated values of blood pressure [8]. Recently, Toba et al. [9] showed that NefR developed hypertension, which does not decrease with the administration of the substrate of NO synthase (NOS), L-arginine.

On the other hand, it is known that models of hypertension, like spontaneously hypertensive rats (SHR) [10], have alterations in basal tone of vascular smooth muscle cells (VSMC). Accordingly, we previously demonstrated that arterial vessels from SHR [11] and coarctation-hypertensive rats [12] showed increased basal tone. This increased basal tone was evidenced by a relaxant response to sodium nitroprusside (SNP) and atrial natriuretic peptide. Other findings from our laboratory, in isolated human arteries, showed an increased basal tone in both hypertensive and normotensive patients [13], indicating that the basal tone of VSMC is not only a consequence of the development of hypertension. Consequently, the integrity of the vascular function and the bioavailability of NO could play a pivotal role in vascular homeostasis and its alteration is involved in an increased basal tone. Another factor that could alter the basal tone is the oxidative stress [14] through inactivation of NO and alteration of vascular reactivity. Moreover, the oxidative stress may also produce a direct vasoconstrictor effect [15] accompanied to an increase of myogenic tone. NADPH oxidase is a source of superoxide anion in the vessel wall. Superoxide anion is involved in increased blood pressure, vascular hypertrophy, and endothelial dysfunction [15, 16] and plays a role in the development of spontaneous vascular tone [11].

In the light of these considerations, the objectives of the present work were to evaluate possible alterations of the endothelial function, NO bioavailability, and basal tone in aortic rings from NefR and establish the role of oxidative stress.

## 2. Methods

### 2.1. Animal Model

In order to obtain a nonischemic model of renal mass reduction, two surgeries were performed to male Sprague Dawley rats: first, the resection of 2/3 of the left kidney (the two poles) and second, after 2 weeks, a complete resection of the right kidney. After each surgery the animals were placed in acclimatized cages until recovery. After 13-14 weeks of second surgery, creatinine clearance (Ccr) (Wiener Lab, Argentina) and microalbuminuria (BioSystem kit, Spain) were measured in metabolic cage by 24 hours. Mean arterial pressure (MAP) was measured by direct method through cannulation of right carotid with a catheter connected to a pressure transductor [12]. In all surgical procedures the animals were anesthetized with sodium pentobarbital (45 mg/kg intraperitoneal). In all cases the rats were sacrificed under anesthesia by exsanguination. The thoracic aorta was dissected, immersed in Krebs solution (in mmol/L: NaCl 122, KCl 5.9, NaHCO_3_ 25, CaCl_2_ 1.9, MgSO_4_ 1.2, and glucose 11), and cut into 5 mm rings (1 to 4 for rat). In some experiments the endothelium was removed (rubbed rings). The rats were divided into 2 groups: one without treatment (NefR, *n* = 8) and other treated with tempol (1 mmol/L in drinking water) since the first surgery until sacrifice (NefR-T, *n* = 6). Results were compared with sham rats (SR: *n* = 7). In SR the two surgeries were performed without removal of any portion of the renal mass.

All experiments were carried out according to the guidelines of the institutional ethics committee.

### 2.2. Contractility

The rings were fixed in an isolated organ chamber with 6 mL of Krebs solution maintained at 37°C, gassed with a mixture of 95% O_2_ and 5% CO_2_ (pH 7.4) [13], and connected to a force transducer (GOULD UC2, USA) and to a recorder (K&Z BD41, Holland). The rings were equilibrated at 2 g of tension by 120 min, which was found to be the optimal tension for KCl-induced contraction (100 mM). KCl-contraction was similar in all groups (SR: 1392 ± 150 mg; *n* = 7; NefR: 1187 ± 131; *n* = 8 and NefR-T: 1028 ± 154; *n* = 6; *P*: NS). Data were expressed in milligrams (mg) of tension.

To evaluate the spontaneous basal tone, the endothelium-independent agent (SNP) was added to rings not previously exposed to any vasoactive agent [12]. For this purpose, SNP was used to maximal dose (10^−5^ M), which was found to be the optimal concentration for SNP-induced relaxation (100%) of the precontracted rings with norepinephrine (NE). 

In some rings, SNP-response was evaluated after incubation (20 min) with N*ω*-nitro-L-arginine methyl ester (L-NAME) 10^−4^ M.

In NefR, in order to evaluate the possible role of oxidative stress in the basal tone, unrubbed aortic rings were subjected to a “preconditioning manoeuvre.” For this purpose some aortic rings were incubated with tempol 10^−5^ M or diphenyliodonium (DPI) 10^−4^ M during 120 min (preconditioned vessels). After that, this agent was removed by washing (40 min) with Krebs solution and SNP stimulation was performed. 

Endothelial function was tested with cumulative dose response curve to acetylcholine (Ach) (10^−9^–10^−5^ M) in NE 10^−5^ M-contracted rings. In all cases the maximal relaxation was induced by Ach 10^−5^ M. Control experiments were performed in rings incubated (20 min) with L-NAME 10^−4^ M. 

### 2.3. Nitrites

Nitrites were measured, by the Griess reaction, in samples from the bath of isolated aortic rings subjected to stretching (2 g) [13]. In some rings, nitrites were measured in the presence (incubation by 20 min) of L-NAME 10^−4^ M, tempol 10^−5^ M, or DPI 10^−4^ M. Data were expressed in pmol/mg of tissue.

### 2.4. Direct NO Measurement

NO release was measured in real time with an ISO-NOP electrode (WPI, USA) connected to a recorder (Apollo 4000, WPI, USA) in isolated aortic rings subjected to stretching [17] at optimal tension for KCl-induced contraction (2 g). Some rings were treated with L-NAME 10^−4^ M or tempol 10^−5^ M. Data were expressed in nanoamperes (nA).

### 2.5. Electrophysiological Studies

Membrane potential (MP) was recorded in VSMC with electrodes connected to an amplifier (WPI, USA) and to a recorder (Gould, USA) [18] in basal conditions and after in vitro treatment with tempol 10^−5^ M. Results were expressed in mV as differences between MP obtained by KCl 100 mM (SR: +7.0 ± 2.7 mV; *n* = 6; NefR: +6.0 ± 1.7; *n* = 6; NefR-T: +6.6 ± 1.2; *P*: NS) and MP registered (basal or tempol). 

### 2.6. Oxidative Stress

Total, reduced (GSH), and oxidized glutathione(GSSG) and thiobarbituric acid reactive substances (TBARS) were determined in aorta homogenates by spectrophotometry [17, 19, 20] and correlated with protein contents.

### 2.7. Statistical Analyses

Results were expressed in mean ± standard error. Statistical analyses were performed with Statistica 5.0 programs. Student's *t*-test for paired samples and ANOVA (One or Two way with Newman-Keuls posttest) were used when appropriate. Results were considered significant when *P* < 0.05.

## 3. Results

Clinical characteristics of the animals are shown in Table 1. Resection of 5/6 of renal mass (NefR) induces an increase of MAP and microalbuminuria, whereas Ccr was decreased. In vivo treatment with tempol (NefR-T) was ineffective in reversing the hypertension but was able to improve Ccr and microalbuminuria (Table 1). Urinary nitrites were higher in SR (417.6 ± 59.2 pmol/mL; *n* = 6) than NefR (94.5 ± 18.5; *n* = 6; *P* < 0.001; One way ANOVA) and NefR-T (227.5 ± 49.0 pmol/mL; *n* = 6; *P* < 0.01; One way ANOVA). NefR-T improves urinary nitrites inrespect to NefR (*P* < 0.05; One way ANOVA).

### 3.1. Endothelial Function

Endothelial function, checked through Ach-vasorelaxant response, was present in SR aortic rings: −796 ± 187 mg (−70 ± 18% of NE-contraction, *n* = 7). A significant decrease of Ach-response was observed in NefR: −70 ± 10 mg (−30 ± 4% of NE-contraction, *n* = 8; *P* < 0.01in respect to SR; One way ANOVA). NefR-T improves endothelial function: the Ach-vasorelaxation was −312 ± 169 (−50 ± 6% of NE-contraction, *n* = 6; *P* < 0.05 in respect to NefR; One way ANOVA). There were not significant differences in Ach-response between SR and NefR-T (*P*: NS; One way ANOVA). In all cases, rubbed manoeuvres or incubation with L-NAME abolished the Ach-response (data not shown). 

### 3.2. Basal Tone

**Figure 1:**
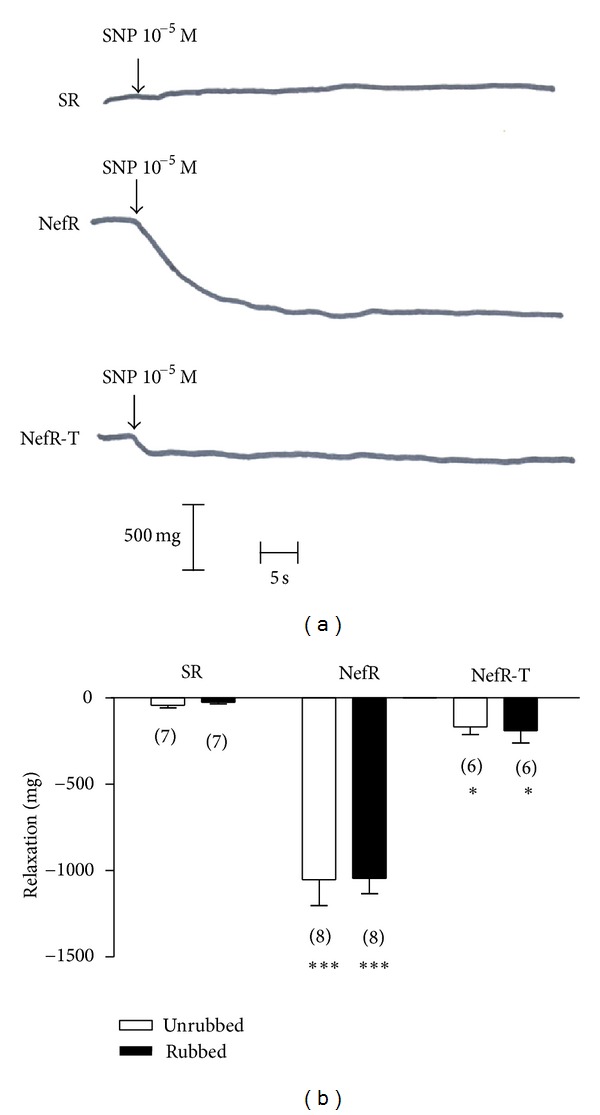
Effect of SNP 10^−5^ M on basal tone. (a) Tracing of a typical experiment of administration of SNP (arrows) on basal tone in unrubbed aortic ring from SR (upper), NefR, (middle) and NefR-T (lower). (b) Average of effect of SNP on basal tone in unrubbed (white bars) and rubbed (black bars) aortic rings from SR, NefR, and NefR-T. ***P < 0.001 NefR versus SR; *P < 0.05 NefR-T versus SR. Two way ANOVA. Data are expressed as mean ± standard error. The number of rings is given in parentheses.

Figure 1(a) shows recorders from typical experiments of the effect of SNP on the basal tone. SNP had no effect on aortic rings from SR (upper panel). However, SNP produced a vasorelaxant response in aortic rings from NefR (middle panel), indicating an increased basal tone. In vivo treatment with tempol (NefR-T) reverted near completely the SNP effect (down panel), indicating a decreased basal tone. The averages of these responses are shown in Figure 1(b) (first group of bars). In any case the rubbed manoeuvres did not modify the effect of SNP (Figure 1(b), second group of bars).

In vitro incubation with L-NAME did not modify SNP-response on basal tone in aortic rings from SR (Krebs: −41 ± 15 mg, *n* = 6 versus L-NAME: −38 ± 15, *n* = 6; *P*: NS), NefR (Krebs: −1052 ± 149 mg, *n* = 8 versus L-NAME: −967 ± 78, *n* = 6; *P*: NS), and NefR-T (Krebs: −168 ± 44 mg, *n* = 6 versus L-NAME: −206 ± 22, *n* = 6; *P*: NS).

On the other hand, in aortic rings from NefR, in vitro preconditioning manoeuvre with antioxidant agents was effective to reverse the increased basal tone. Preconditioning with tempol (10^−5^ M by 120 min) decreased the SNP response. The effect was −244 ± 83 mg (−77 ± 8% from baseline, *n* = 6; *P* < 0.01). Similar effect was obtained by preconditioning with DPI (10^−4^ M by 120 min): −87 ± 3% from baseline; *n* = 6; *P* < 0.01. 

### 3.3. Nitrite Levels

**Figure 2:**
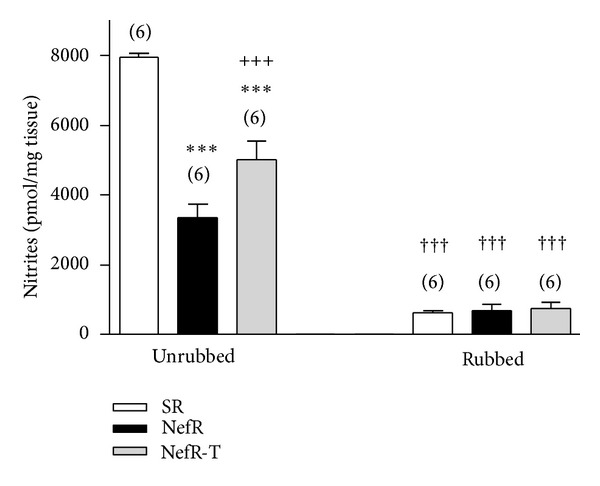
Nitrite contents in aortic rings of SR (white bars), NefR (black bars), and NefR-T (gray bars) with (first group of bars: unrubbed) or without functional endothelium (second group of bars: rubbed) at 15 min in the experiment. ***P < 0.001 NefR and NefR-T versus SR; ^+++^ *P* < 0.001 NefR-T versus NefR; ^†††^ *P* < 0.001 rubbed versus unrubbed rings. Two way ANOVA. Data are expressed as mean ± standard error. The number of rings is given in parentheses.

Figure 2 shows nitrite levels in aortic rings. Nitrite levels were higher in SR than NefR. NefR-T improves the nitrite levels. In all cases rubbing manoeuvres decreased nitrites (Figure 2).

In the presence of L-NAME 10^−4^ M, nitrite levels were decreased in unrubbed rings from SR (−90 ± 2% from baseline; *n* = 6; *P* < 0.001; paired Student's *t*-test), NefR (−75 ± 10% from baseline; *n* = 6; *P* < 0.001; paired Student's *t*-test), and NefR-T (−92 ± 5% from baseline; *n* = 6; *P* < 0.001; paired Student's *t*-test). 

In NefR aortic rings, in vitro administration of tempol increased nitrite levels. The nitrite values in the presence of tempol 10^−5^ M were 6369 ± 433 pmol/mg (*n* = 6; 89 ± 13% increase over baseline; *P* < 0.01; paired Student's *t*-test). Similar results were observed with in vitro administration of DPI 10^−4^ M (73 ± 14% over baseline, *n* = 7; *P* < 0.01; paired Student's *t*-test). However, in SR and NefR-T these agents did not modify nitrites.

**Figure 3:**
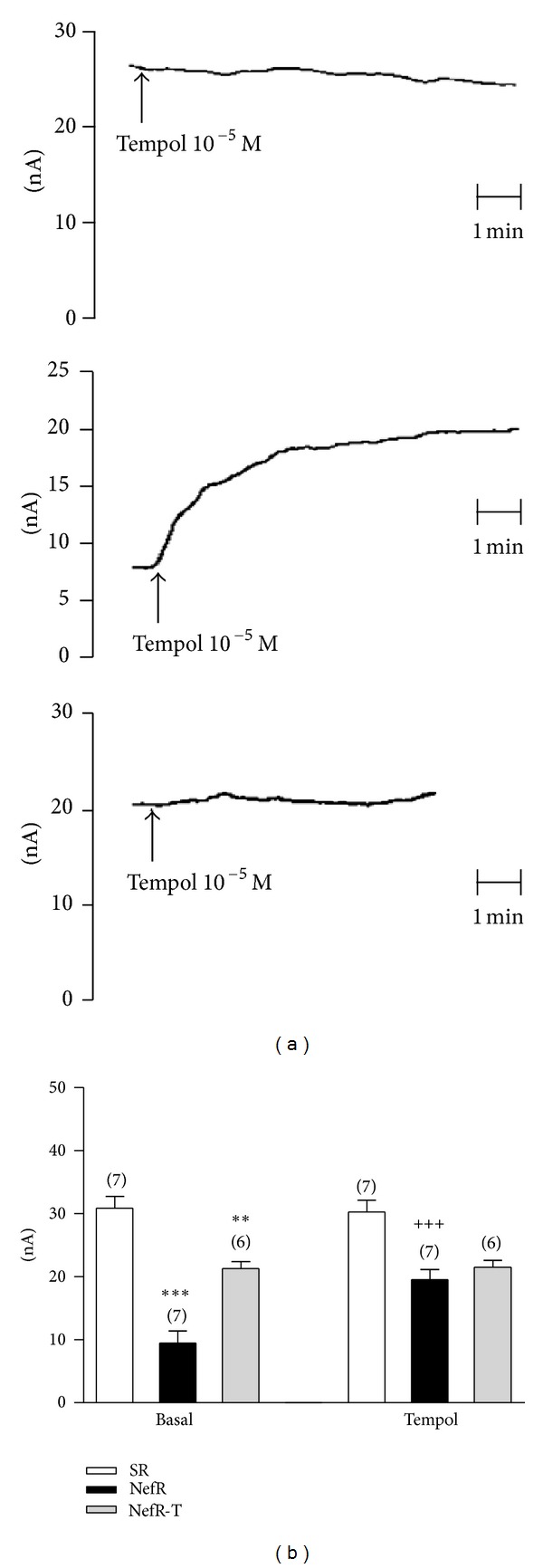
Effect of tempol 10^−5^ M on NO levels (a) Typical experiment of direct measurement of NO in unrubbed aortic rings from SR (upper), NefR (middle), and NefR-T (lower) and the effect of tempol (arrows). (b) Average of effect of tempol on direct measurement of NO in unrubbed aortic rings of SR (white bars), NefR (black bars), and NefR-T (gray bars). **P < 0.01 NefR-T versus SR; ***P < 0.001 NefR versus SR; ^+++^ *P* < 0.001 tempol versus basal. Two way ANOVA. Data are expressed as mean ± standard error. The number of rings is given in parentheses.

Figure 3 shows direct NO measurement in SR, NefR, and NefR-T and the effect of in vitro administration of tempol.  Figure 3(a) shows recorders from typical experiments of the effect of tempol on NO release in SR (upper panel), NefR (middle panel), and NefR-T (bottom panel).  Figure 3(b) shows the average of these responses. First group of bars shows the NO levels in basal conditions. NO release was higher in SR than NefR. NefR-T improves the NO release. Tempol had no effect in SR and NefR-T; however it was able to increase NO in NefR (Figure 3(b), second group of bars). 

In vitro administration of L-NAME was able to decrease NO release in SR (−87 ± 1% from baseline; *n* = 6; *P* < 0.001; paired Student's *t*-test) and NefR (−64 ± 1% from baseline; *n* = 8; *P* < 0.01; paired Student's *t*-test). Similar inhibition was obtained in NefR-T (data not shown). 

### 3.4. Membrane Potential

**Figure 4:**
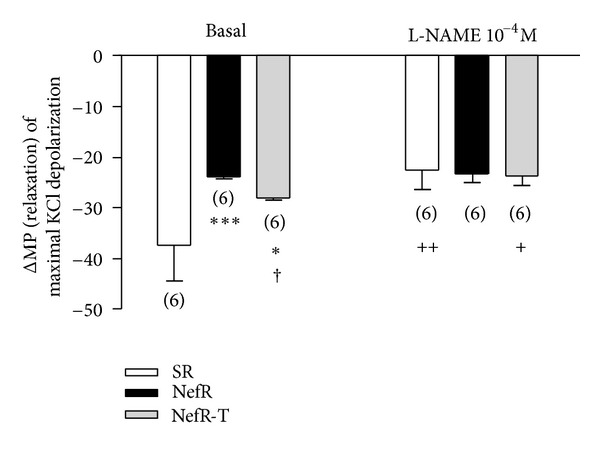
Membrane potential (MP) in unrubbed aortic rings from SR (white bars), NefR (black bars), and NefR-T (gray bars) in basal conditions (Krebs) and the effect of administration of L-NAME 10^−4^ M. ***P < 0.001 NefR basal versus SR basal; *P < 0.05 NefR-T basal versus SR basal; ^++^SR L-NAME *P* < 0.01 versus SR basal; ^+^ *P* < 0.05 NefR-T L-NAME versus NefR-T basal; ^†^ *P* < 0.05 NefR-T basal versus NefR basal. Two way ANOVA. Data are expressed as mean ± standard error. The number of rings is given in parentheses.

Figure 4 shows the MP in basal conditions and after administration of L-NAME in unrubbed aortic rings from SR, NefR, and NefR-T. Basal MP was higher in NefR than SR. NefR-T partially recovered MP values (Figure 4, first group of bars). In vitro treatment with L-NAME produced a partial depolarization in SR and NefR-T but not in NefR (Figure 4, second group of bars). 

In vitro administration of tempol induced a hyperpolarizing effect only in NefR (MP: −30 ± 1 mV; 124 ± 5% from baseline; *n* = 6; *P* < 0.01 paired Student's *t*-test).

### 3.5. Oxidative Stress

In aortic rings, GSH levels were higher in SR (33.9 ± 7.2 *μ*mol/mg protein; *n* = 6) than NefR (3.8 ± 1.4; *n* = 6; *P* < 0.001). However, similar GSSG levels were found in SR (5.6 ± 0.6 *μ*mol/mg protein; *n* = 6) and NefR (5.2 ± 0.3; *n* = 6; *P*: NS). In agreement, the GSH/GSSG ratio was higher in SR (6.3 ± 1.0; *n* = 6) than NefR (0.7 ± 0.2; *n* = 6; *P* < 0.001; One way ANOVA). NefR-T improves GSH/GSSG (data not shown).

In aortic rings, TBARS levels were higher in NefR (5.2 ± 0.7 nmol/mg protein; *n* = 6) than SR rings (1.5 ± 0.3; *n* = 6; *P* < 0.001; One way ANOVA). NefR-T decreased the TBARS levels (1.2 ± 0.6 nmol/mg protein; *n* = 6; *P* < 0.001 versus NefR; One way ANOVA). No significant differences were found between SR and NefR-T in TBARS levels (*P*: NS; One way ANOVA).

## 4. Discussion

The novel finding of this study is that (1) subtotal nephrectomy induces an associated hypertension with an increased basal tone, an endothelial dysfunction, and an oxidative state and that (2) in vivo treatment with tempol improves arterial basal tone, NO levels, and oxidative stress without reversing hypertension, indicating that oxidative stress could be implicated in the vascular alterations, and that these disorders would occur early and independently to hypertension.

It has been demonstrated that several models of renal mass reduction induce CKD and alter the renal function [6, 21, 22]. On the other hand, CKD is frequently associated with hypertension [23, 24]. A lot of evidence has reported that the decreased renal mass is a cardiovascular risk factor [2]. In experimental models, some authors have reported that the loss of renal mass induces hemodynamic alterations that lead to hypertension [4]; however the physiopathological mechanisms involved remain unclear. It is known that an endothelial function and an NO bioavailability play a pivotal role in the balance of vascular tone. Likewise, an increase in basal tone may be associated with the presence of oxidative stress. In a model of CKD with loss of renal mass associated with renal ischemia, it has been shown that oxidative stress is implicated in the hypertension developed [25]. In our work, nonischemic 5/6 nephrectomized rats showed hypertension associated with an impaired endothelial function and a decreased NO levels. In agreement with this, other authors found that the vessels from nonischemic 5/6 nephrectomized rats have a decreased Ach-response indicating an endothelial dysfunction [26]; however, these alterations were developed in the absence of hypertension. 

In relation to the increased basal tone in NefR, previous reports from our laboratory have shown an increased basal tone in coarctation-hypertensive rats [12] and SHR [11]. Also, we were able to increase the basal tone by an “in vitro” sensitizing manoeuvre in vessels from rabbits [27] and toads [28]. For this purpose, normotensive aortic rings were previously sensitized with a vasoconstrictor agent, washed with Krebs by 120 min, and then stimulated with a vasorelaxant agent. On the other hand, it is known that oxidative stress could alter the basal tone. In this sense, in our laboratory, we found that the basal tone of SHR was increased by oxidative stress [11]. Also, it was reported that the oxidant agent H_2_O_2_ could alter vascular contractility in normotensive and hypertensive rats [29]. This is in agreement with the finding from a present report, in which antioxidant treatment decreased the basal tone in aortic rings from NefR. An other result from present work that supports the fact that vessels of NefR presented an increased oxidative stress is the altered GSH/GSSG ratio and increased TBARS levels. 

Despite that NefR and NefR-T showed similar values of MAP, the aortic rings from NefR-T decreased basal tone, evidenced by a lower SNP response. This fact could indicate that in vivo treatment with antioxidant agents may decrease basal tone independently of the blood pressure values. At variance with our findings, in other model of CKD associated with renal ischemia [25], tempol decreased MAP values. This difference may be explained since renal ischemia is not present in our model of CKD and, it is known that renal ischemia is strongly associated with great activation of renin-angiotensin system in which angiotensin II (Ang II) not only has hemodynamic effects but also has tissue actions, which results in an increase of oxidative stress. Accordingly, some works have demonstrated that Ang II activation of NADPH increases reactive oxygen species [30]. However, in our model these additional effects of Ang II on oxidative stress are not present. 

The role of endothelium in the vascular function and its association with CKD are well known. In present work we found that NefR showed an endothelial dysfunction with decreased NO levels. The fact that in vivo treatment with tempol improves endothelial function and NO bioavailability indicates that the endothelium damage is produced, at least in part, by oxidative stress. 

In our work we found that NefR decreased urinary nitrites. Similar findings have beenreported by other authors, who also found reduced renal NO-synthases in a similar model of subtotal nephrectomy [7]. Unlike other works performed in models of subtotal nephrectomy, in our study we measured nitrite and NO in vessels. We observed that the decreased vascular NO levels were increased by tempol (in vivo and in vitro), indicating a role of oxidative stress in the vascular NO bioavailability. In accordance, it has been reported that superoxide dismutase improves NO-dependent vasorelaxation in other models of CKD [25]. 

Assuming that the oxidative stress decreases NO levels and increases the basal tone [11], we could hypothesize that antioxidant treatment improves NO bioavailability and also decreases basal tone. In fact, in vitro administration of tempol improves NO bioavailability and reduces the SNP response in aortic rings. Moreover, the in vivo treatment with antioxidant agents (NefR-T) showed similar results. 

In conclusion, in the present work we described, for the first time, that aortic rings from nonischemic 5/6 nephrectomized rats presented an increased basal tone related to endothelial dysfunction and decreased NO levels. The fact that in vitro and in vivo treatments with tempol improve the NO bioavailability and the basal tone, without decreasing the values of blood pressure, indicates that oxidative stress could be implicated in the vascular alterations and that these disorders would occur early and independently to hypertension.

## Figures and Tables

**Table 1:** Clinical characteristics of rats.

	Rat		
	SR (*n* = 7)	NefR (*n* = 8)	NefR-T (*n* = 6)
Body weight (g)	226.3 ± 8.4	218.5 ± 4.5	220.5 ± 5.2
Food intake (g/24 hs)	24.7 ± 3.8	18.2 ± 1.8	23.0 ± 3.6
Water intake (mL/24 hs)	22.7 ± 1.6	41.6 ± 4.1***	40.8 ± 2.6^†††^
Urinary volume (mL/24 hs)	10.7 ± 2.2	28.1 ± 3.7***	23.5 ± 2.4^++^
Glucose (mg/dL)	80.0 ± 0.1	83.2 ± 1.3	82.1 ± 1.1
Mean arterial pressure (mm Hg)	119 ± 4	169 ± 6***	168 ± 10^†††^
Heart rate (beats/min)	365 ± 21	353 ± 16	354 ± 15
Creatinine clearance (mL/min)	0.59 ± 0.1	0.11 ± 0.01***	0.22 ± 0.04^††,+^
Microalbuminuria (mg/24 hs)	6.8 ± 1.2	62.9 ± 9.8***	22.1 ± 5.2^†,+++^

SR: sham rats; NefR: nefrectomized rats; NefR-T: NefR treated with tempol; ***P < 0.001 NefR versus SR; ^†^ *P* < 0.05, ^††^ *P* < 0.01, and ^†††^ *P* < 0.001 NefR-T versus SR; ^+^ *P* < 0.05, ^++^ *P* < 0.01, and ^+++^ *P* < 0.001 NefR-T versus NefR. One way ANOVA.
